# The Benefit of Autonomy and Control: A Positive Characteristic of the Home Health Aide Position

**DOI:** 10.1093/geroni/igab046.936

**Published:** 2021-12-17

**Authors:** Hayley Gleason, Edward Miller

**Affiliations:** 1 Colorado Department of Health Care Policy & Financing, Denver, Colorado, United States; 2 University of Massachusetts Boston, Boston, Massachusetts, United States

## Abstract

Home Health Aides’ (HHAs) are one of the fastest growing workforces in the country, yet the industry struggles to recruit new aides into the field and retain current workers. This study explored HHAs’ experiences with the level of autonomy and control granted to them within their day-to-day work. Findings from six focus groups with 37 HHAs showed that many aides select home care because of the control and independence the positions offer. Interacting one-on-one with clients and being able to self-structure their daily tasks were major benefits that drew HHAs to the field. Additionally, the HHAs highlighted the control they have over their schedule and the flexibility the position offers to enable them to accommodate other responsibilities, like childcare or other jobs. Being able to decline a client because of travel distance, the hours required, or not feeling that it is a “good fit” was also a welcomed aspect of the position. Despite complaints about the job, such as low pay, lack of benefits, and limited support, many of the HHAs admitted staying on in their positions because of the flexibility, autonomy, and control provided. Findings highlight the value that HHAs place on autonomy and control and the potential benefit that these job qualities have for promoting greater recruitment and retention of the home care workforce. Amplifying opportunities for these aspects of the job may thus entice new individuals to pursue a career as an HHA, as well as help to maintain those individuals currently in the position.

